# Uterine Cytokine Production During the Menstrual Cycle and Preimplantation Stages in Mice

**DOI:** 10.1155/1999/67137

**Published:** 1999

**Authors:** Irene Athanassakis, Vagia Farmakiotis, Lina Papadimitriou

**Affiliations:** 1 Department of Biology University of Crete Heraklion Crete Greece; 2 Department of Biology University of Crete P.O. Box 1470 Heraklion Crete 711-10 Greece

**Keywords:** Uterus, cytokines, menstrual cycle, pregnancy

## Abstract

Female reproduction is the only system subjected to well defined periodic changes. The final stage of the menstrual cycle in mammals is the maturation of the ovum and the preparation of the female organism to support fetal development fertilization. Once pregnancy occurs, both maternal and fetal sites emit regulatory signals to ensure embryo development and maternal protection against a graft versus host (GvH) reaction initiated by the semi-allogeneic fetus. We and others have previously shown that each day of fetal development in mice is characterized by different cytokine production, detected not only at the proximity of the feto-placental unit (decidua, uterus), but also in maternal lymphoid organs (spleen), as well as in the serum. In the present study, we concentrated on the menstrual cycle and the preimplantation stages of pregnancy and defined the levels of GM-CSF, IL-10, IL-6, and IL-3 in the murine uterus during anoestrus, proestrus, oestrus, and second and third day of gestation. We show by immunofluorescence and ELISA techniques that GM-CSF is maintained at high levels during anoestrus, proestus, oestrus, and the second day of pregnancy while dropping on the third day. IL-3 levels are found elevated during proestrus, second and third day of gestation, IL-6 increases essentially during proestrus, whereas the production of IL-10 was detected during oestrus and the early stages of pregnancy. Immunoperoxidase staining on frozen sections of uteri during the early gestational period localize GM-CSF and IL-3 production in the endometrium, IL-10 in the endometrium on the second day of pregnancy, and endometrium/myometrium on the third day. Low levels of IL-6 could be detected in the endometrium/epithelium on the second day and endometrium/myometrium on the third day of gestation. The role of IL-3, IL-10, and, to a lesser degree, IL-6 is fortified by the embryo itself, since these cytokines were found to be produced by blastocysts as well. These results demonstrate the existence of a specific distribution of lymphokines within the uterine tissue, the role of which is being discussed.


*Developmental Immunology*, 1999, Vol. 7(1), pp. 33-42
Reprints available directly from the publisher
Photocopying permitted by license only

© 1999 OPA (Overseas Publishers Association)
N.V. Published by license under the
Harwood Academic Publishers imprint,
part of Gordon and Breach Publishing Group
Printed in Malaysia

### Uterine Cytokine Production During the Menstrual Cycle and Preimplantation Stages in Mice

IRENE ATHANASSAKIS*, VAGIA FARMAKIOTIS and LINA PAPADIMITRIOU

*Department of Biology, University of Crete, Heraklion, Crete, Greece*

*(Received 20 May, 1998; In final form 30 October, 1998)*

Female reproduction is the only system subjected to well defined periodic changes. The final stage of the menstrual cycle in mammals is the maturation of the ovum and the preparation of the female organism to support fetal development fertilization. Once pregnancy occurs, both maternal and fetal sites emit regulatory signals to ensure embryo development and maternal protection against a graft versus host (GvH) reaction initiated by the semi-allogeneic fetus. We and others have previously shown that each day of fetal development in mice is characterized by different cytokine production, detected not only at the proximity of the feto-placental unit (decidua, uterus), but also in maternal lymphoid organs (spleen), as well as in the serum. In the present study, we concentrated on the menstrual cycle and the preimplantation stages of pregnancy and defined the levels of GM-CSF, IL-10, IL-6, and IL-3 in the murine uterus during anoestrus, proestrus, oestrus, and second and third day of gestation. We show by immunofluorescence and ELISA techniques that GM-CSF is maintained at high levels during anoestrus, proestus, oestrus, and the second day of pregnancy while dropping on the third day. IL-3 levels are found elevated during proestrus, second and third day of gestation, IL-6 increases essentially during proestrus, whereas the production of IL-10 was detected during oestrus and the early stages of pregnancy. Immunoperoxidase staining on frozen sections of uteri during the early gestational period localize GM-CSF and IL-3 production in the endometrium, IL-10 in the endometrium on the second day of pregnancy, and endometrium/myometrium on the third day. Low levels of IL-6 could be detected in the endometrium/epithelium on the second day and endometrium/myometrium on the third day of gestation. The role of IL-3, IL-10, and, to a lesser degree, IL-6 is fortified by the embryo itself, since these cytokines were found to be produced by blastocysts as well. These results demonstrate the existence of a specific distribution of lymphokines within the uterine tissue, the role of which is being discussed.

*Keywords:* Uterus, cytokines, menstrual cycle, pregnancy

### INTRODUCTION

The progression of events from ovum maturation to fertilization and completion of pregnancy is highly regulated by sex steroid hormones, cytokines, as well as neuropeptides. The involvement of oestrogens, follicule stimulating hormone (FSH), luterizing hormone (LH), progesterone to ovum maturation, embryo

* Corresponding author. Department of Biology, University of Crete, P.O. Box 1470, 711–10 Heraklion, Crete, Greece. Tel. #: +30 (081) 39.43.55, Fax #: +30 (081) 39.44.08. E-mail : athan@biology.uch.gr implantation, and maintenance of pregnancy are undisputable events, yet their action is still subject of intense investigation.

It has been demonstrated that many cytokines play important roles during the different stages of reproduction. Concentrating our interest on ovum maturation and early pregnancy events, we can mention the inhibitory role of tumor necrosis factor-α (TNF-α) to steroid biosynthesis, sperm mobility, and sperm/cervical mucus interactions (Hill and Anderson, 1991). IFN-γ has also been reported to inhibit sperm mobility (Hill and Anderson, 1989), whereas IL-1β and CSF-1 are acting as implantation inhibitors during early pregnancy (Haimovici et al., 1991; Tartakovsky et al., 1991). Moreover, the involvement of neuropeptides such as cortico-releasing hormone (CRH) and β-endorphin has also been postulated to monitor embryo implantation (Makrigiannakis et al., 1995). It appears thus that the study of these and other regulatory pathways is mandatory in order to understand the fine reactions taking place in the uterus during the menstrual cycle and early pregnancy.

In this paper, we study the intrauterine involvement of granulocyte-macrophage colony stimulating factor (GM-CSF), interleukin-3 (IL-3), interleukin-10 (IL-10), and interleukin-6 (IL-6) during the menstrual cycle and postconceptional days of pregnancy in mice. Although GM-CSF, IL–3, and IL-10 are known to emit positive regulatory signals to trophoblasts and late embryo development (Athanassakis et al., 1987; Chaouat et al., 1990, 1995), their role in early stages is debatable. We show that although a high percentage of uterine cells synthezise GM-CSF during the menstrual cycle, this cytokine is not secreted in high amounts until the third day of pregnancy. Intracellular IL-3 is expressed essentially during proestrus and the third gestational day, but its secretion can only be detected as pregnancy begins, reaching higher levels on the third day. Secreted IL-10 can be detected after the initiation of the gestational cycle, yet it can also be detected intracellularly during oestrus. IL-6 is considered to be an inflammatory cytokine, harmful to the late embryo (Heinrich et al., 1990), but possibly necessary to initiate decidualization in the uterus. We show that although IL-6 is intracellularly produced during proestrus, it is secreted only in low amounts on day 3 of pregnancy when implantation is expected to take place. Immunohistological studies localize GM-CSF and IL-3 positive cells until the fourth and third day of pregnancy, respectively, in the endometrium, IL-10 in the endometrium/myometrium, whereas IL-6 can be detected sparsely into endometrium, myometrium, as well as uterine epithelium. IL-3, IL-10 and IL-6 production is also supported by the embryonic counterpart since these cytokines were also found to be produced by blastocysts.

### RESULTS

Since the pattern of cytokine production during the menstrual cycle and the first days of pregnancy is essential to the female reproductive system, in the present work, we concentrated on GM-CSF, IL-3, IL-10, and IL-6 and determined the presence of intracellular as well as secreted forms of these cytokines during the menstrual cycle and the first days of pregnancy.

Thus, uteri from BALB/c females were isolated and cultured for one to five days in order to determine the best timing for lymphokine detection. Intracellular immunofluorescence experiments showed that on the third day of culture, all tested lymphokines were reaching maximal levels (Fig. 1), permitting thus to concentrate on this day of culture for the rest of the study. Five different stages of the reproductive cycle were tested: anoestrus, proestrus, oestrus, and second and third day of pregnancy. Cytokine production by uterine cells was estimated by intracellular immunofluorescence, whereas their secretion was determined by indirect ELISA on the uterine cell culture supernatants. Localization of the cytokines within the uterine tissue was estimated by immunoperoxidase staining of frozen uteri sections on days 2 to 5 of pregnancy.

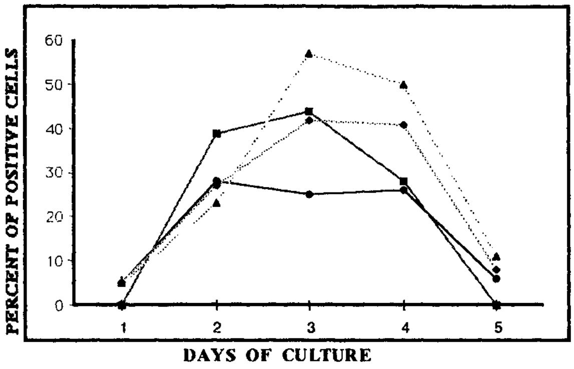

FIGURE 1 Determination of maximal cytokine production during uterine cell cultures. Uterine cells were cultured for various time intervals and intracellular cytokine production of GM-CSF (circle), IL-3 (rectangle), IL-10 (triangle), and IL-6 (rhombus) was determined by immunofluorescence. The results represent the mean of five different experiments performed using three mice every time. In all cases, standard deviation varies from 2 to 6%.

### GM-CSF

Higher production of this factor by the uterine cells is seen during the menstrual cycle since 39–54% of the cells produce GM-CSF, whereas this percentage drops to 24 and 32% during the second and third day of pregnancy, respectively (Fig. 2). Determination of the extracellular presence of the cytokine reveals a specific regulation between synthesis and secretion. Only a small amount of GM-CSF is detected during anoestrus, which increases to 35 and 30% over background during proestrus and oestrus, respectively. Although on the second day of pregnancy the presence of GM-CSF in the culture medium remains at low levels (19% of increase over background), it remarkably reaches 99% of increase during the third day (Fig. 3). Immunoperoxidase staining on frozen sections of uteri localized GM-CSF in the endometrium on the second, third, and fourth day of gestation (Fig. 4).

### IL-3

The highest percentage of IL-3-secreting cells during the menstrual cycle is detected on the stage of proestrus (42%), whereas 54% of the cells produce this cytokine on the third day of pregnancy (Fig 2). However, the positive cells during proestrus do not seem to secrete IL-3 since this factor could not be detected in the culture supernatants in none of the stages of the menstrual cycle. An increasing amount of the factor is secreted after pregnancy begins, reaching 117% of increase over background on the third day of pregnancy (Fig. 3), which correlates with immunoperoxidase staining experiments when IL-3 is detected in the endometrium on days 2 and 3 of pregnancy (Fig. 4).

### IL-6

IL-6-producing cells were essentially detected during proestrus (Fig. 2), which, however, could not be detected in culture supernatants (Fig. 3). This factor was present at low levels (20% of increase over background) in the culture supernatants of uteri on the third day of pregnancy. Immunoperoxidase stainings detected smaller amounts of IL-6 in the endometrium/epithelium on the second day, in the endometrium/myometrium on the third and fourth days, and endometrium on the fifth day of pregnancy (Fig. 4).

### IL-10

The percent of IL-10-producing cells enhances during oestrus and follows an increasing phase after pregnancy occurs (Fig. 2), whereas secretion of the factor is following a similar pattern (Fig. 3). IL-10 was localized in the endothelium during the second day of pregnancy and endometrium/myometrium during the third and fifth day of gestation (Fig. 4).

### Blastocyst Cytokine Production

If the cytokines studied here are important to implantation, their uterine production should be enhanced by fetal components as well. We therefore examined whether blastocysts ensure production of these factors by themselves, so that implantation may be facilitated. Immunofluorescence experiments on whole

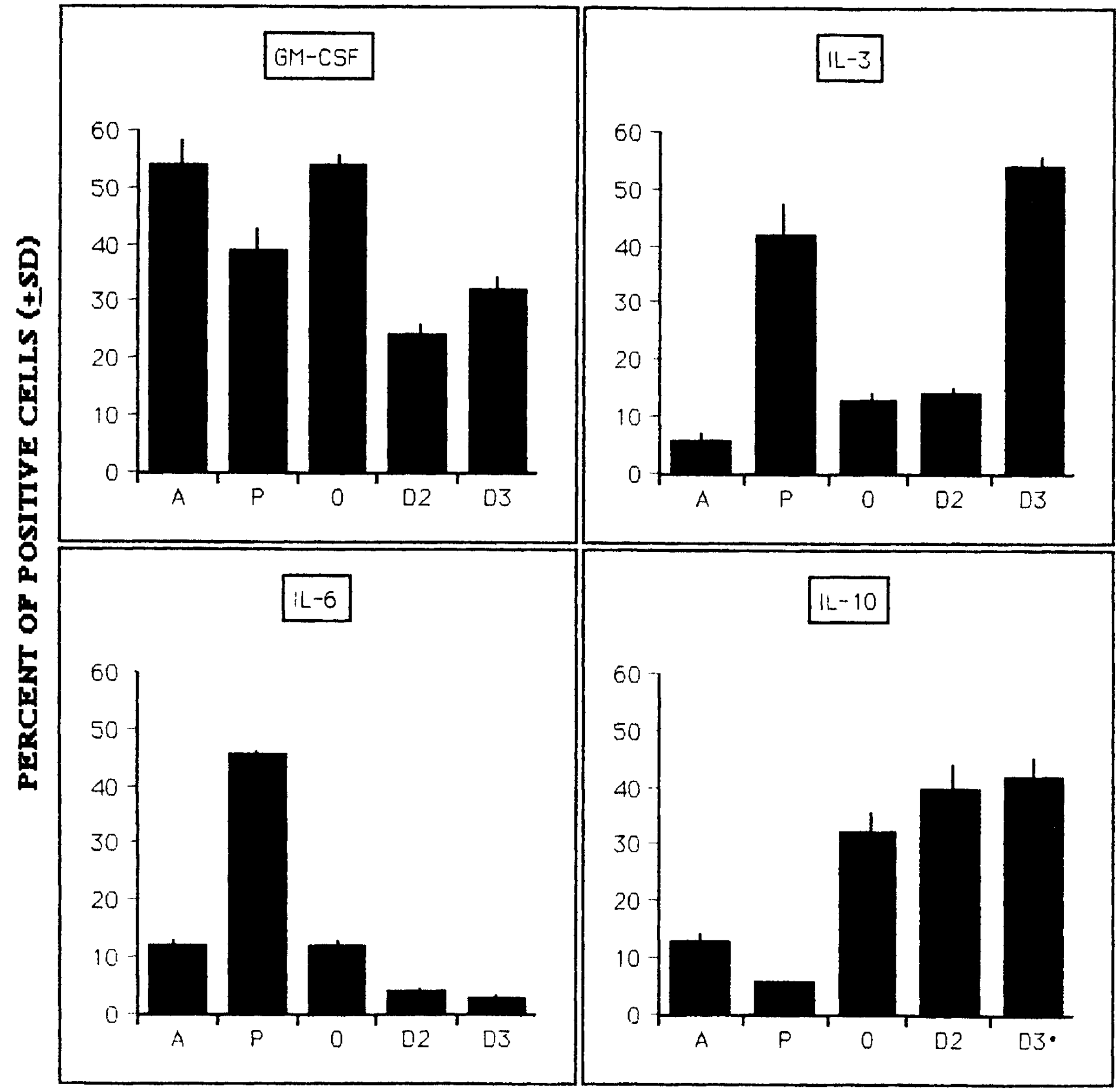

FIGURE 2 Intracellular detection by immunofluorescence of GM-CSF, IL-3, IL-6, and IL-10 in cultured uterine cells during anoestrus (A), proestrus (P), oestrus (O), days 2 and 3 of pregnancy (D2 and D3, respectively). The results represent the mean of 4 experiments and are expressed as the net percentage of positive cells (background values, which varied from 3 to 7%, have been subtracted) ± SD

blastocysts revealed the presence of IL-3 in trophectoderm and IL-10 in trophectoderm and inner cell mass (Fig. 5). Low amounts of IL-6 were detected in the trophectoderm, whereas GM-CSF could not be detected neither in the trophectoderm nor inner cell mass (data not shown).

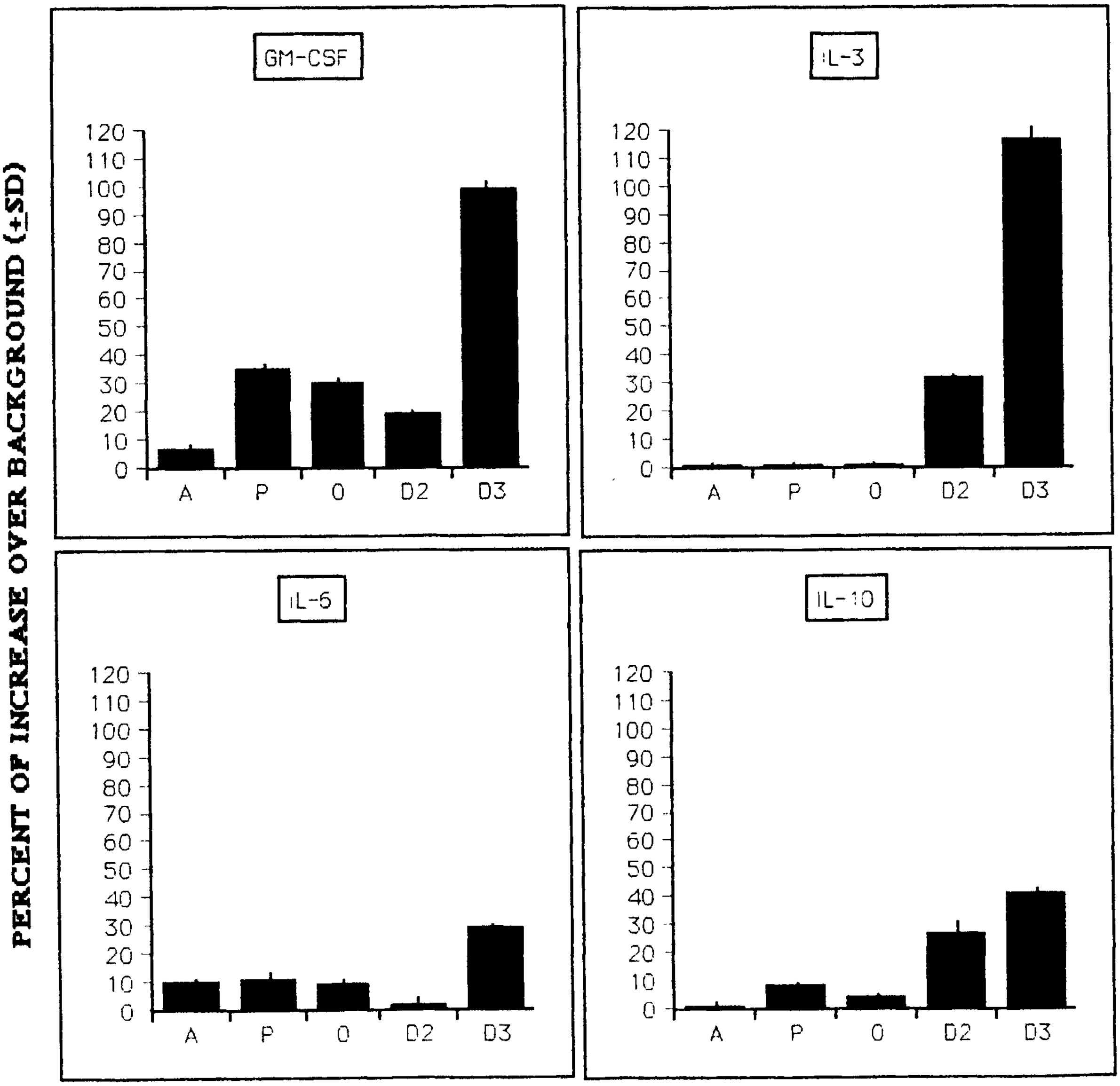

FIGURE 3 Secretion of GM-CSF, IL-3, IL-6, and IL-10 in uterine cell culture medium during anoestrus (A), proestrus (P), oestrus (O), days 2 and 3 of pregnancy (D2 and D3, respectively) as determined by ELISA. The results represent the mean of 4 experiments and are expressed as the percent of optical density increase over background (O D values obtained in the absence of culture supernatants) ± SD

### DISCUSSION

It has become obvious that a number of cytokines are involved in the regulation of the female reproductive system. However, this system is not easily accessible for an in depth study since it is submitted to continuous changes during the menstrual cycle and the gestational period as well. In the present paper, we define the profile of GM-CSF, IL-3, IL-6, and IL-10 in the murine uterus during the menstrual cycle and the first

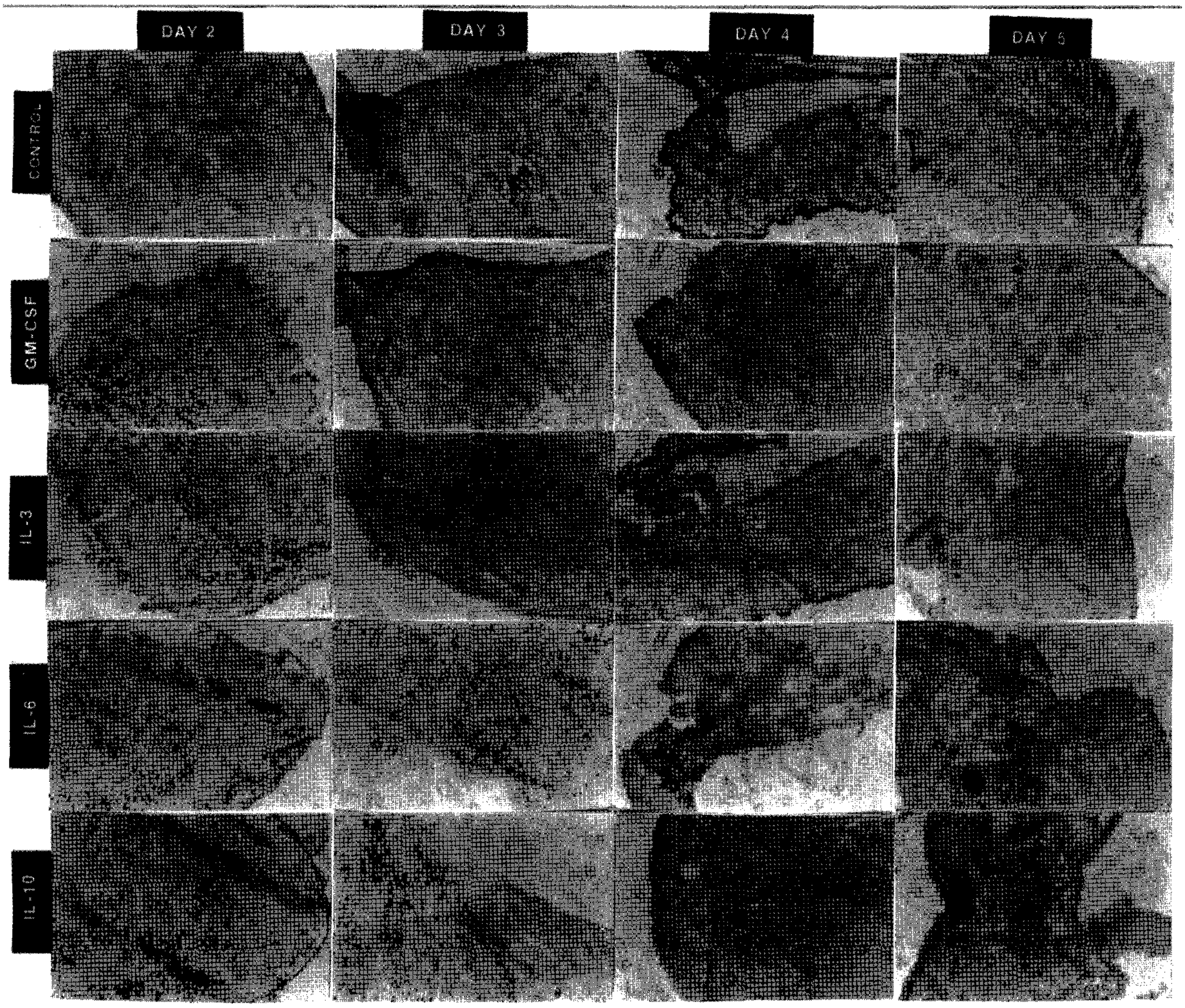

FIGURE 4 Localization of GM-CSF, IL-3, IL-6, and IL-10 in uterine tissue frozen sections on days 2 to 5 of pregnancy by immunoperoxidase staining. Microphotographs × 10

postconceptional days. During the midgestational period, GM-CSF and IL-3 have been shown to improve fetal development by fortifying the placental barrier (Athanassakis et al., 1987), whereas IL-10 has been reported to rescue fetal rejection in experimental abortion models (Chaouat et al., 1995). Except for these three cytokines, which are positively involved in fetal development, we have included in our study IL-6, which is an inflammatory cytokine having harmful effects at mid gestational stages, but not studied for its effects during implantation. Following immunofluorescence staining on uterine cells and ELISA on the supernatants of the uterine cell cultures, we were able to determine the intracellular production and secretion of the cytokines studied. Immunoperoxidase staining on frozen uterine tissue section localized the specific cytokine production from the second to the fifth day of pregnancy.

We detected the intracellular presence of GM-CSF, IL-3, IL-6, and IL- 10 during anoestrus, proestrus,

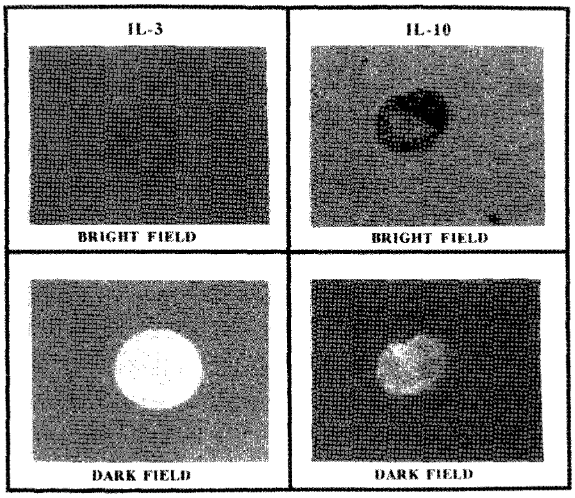

FIGURE 5 Detection of IL-3 and IL-10 in blastocysts by immunofluorescence staining. Microphotographs × 40

oestrus as well as the second and third days of pregnancy. The results showed that GM-CSF was produced by a significant percentage of cells especially during the menstrual cycle, IL-3 was essentially detected during proestrus and the third day of pregnancy, IL-10 followed an increasing pattern from oestrus to the third day of gestation, whereas significant amounts of IL-6–positive cells were detected during proestrus. However, these intracellularly localized factors were not uniformly secreted in the culture medium. The secretion of GM-CSF only on the third day of pregnancy may indicate a probable important role of this factor to blastocyst maturation. Other investigators have shown that *in vivo* administration of GM-CSF induces a high proportion of morulae, but does not sustain normal implantation *in vitro* (Tartakovsky and Ben-Yair, 1991). In order to determine the duration of GM-CSF production, we immunostained frozen sections of uteri from days 2 to 5 of pregnancy and showed that GM-CSF levels decline on the fourth and cannot be detected on the fifth day, indicating that this factor is not needed until later in the pregnancy (Athanassakis et al., 1987; Clark et al., 1994; Garcia-Lloret et al., 1994).

The intracellular IL-3 observed during the menstrual cycle is not secreted since it is undetectable in the culture supernatants, indicating that it may be involved in other intracellular pathways. Indeed, once pregnancy begins, the amount of secreted IL-3 increases, reaching high levels on the third day. In contrast to GM-CSF, immunoperoxidase staining on frozen uterine sections cannot detect IL-3 on the fourth or fifth day of pregnancy, indicating thus a possible necessity of the factor for blastocyst maturation and implantation. The *in situ* experiments localize GM-CSF and IL-3 production in the endometrium. The observed higher amounts of the two cytokines seen on day 2 of pregnancy, instead of day 3 as expected from the ELISA and immunofluorescence experiments, can be explained because on the second day of pregnancy, GM-CSF and IL-3 are sparcely produced by endometrial cells, whereas on the third day, they are accumulated to implantation sites.

A similar analysis for IL-10 shows that only a low amount of the intracellular factor is secreted essentially during the first days of pregnancy. Immunohistological analysis confirmed these results and showed, in addition, the presence of this factor on the fifth day of pregnancy. It therefore seems that except for its protective role later in pregnancy (Chaouat et al., 1995), IL-10 is required during the early stages of fetal development as well. Despite the intracellular localization of IL-10, the secretion of this factor is less spectacular, indicating that the production and secretion of the protein are regulated by different intracellular mechanisms.

The inflammatory cytokine IL-6 is considered harmful to embryo development since it may initiate maternal antifetal immune response leading to fetal rejection. However, one would expect to find IL-6 positively involved during early pregnancy to facilitate the local inflammatory reaction of implantation. The high levels of intracellular IL-6 detected during proestrus are in agreement with previous studies by Prahala and Wira (1995), who showed that IL-6 facilitates maximal antigen presentation ability of the female tract mucosal tissues during this stage of the menstrual cycle. Immunohistological staining detects IL-6 in all stages of pregnancy tested, showing a possible role of this factor during the early gestational stage. Despite the intracellular production, as in the case of IL-10, IL-6 could be detected at low levels only in the culture medium of day 3 pregnant uteri.

Immunofluorescence staining of blastocysts detect IL-3 and IL-6 in trophectoderm, IL-10 in trophectoderm and inner cell mass, whereas GM-CSF could not be found in any of the two populations, indicating that uterine production for at least IL-3, IL-10, and IL-6 is enhanced by the embryo itself, thus encouraging maternal/fetal cross-talk.

This work defines the existence of specific cytokine patterns during the menstrual cycle and the first days of pregnancy in support of fetal development. Studying the physiology of these early reproductive stages is very useful to understanding many cases of infertility.

### MATERIALS AND METHODS

### Animals and Tissue Isolation

BALB/c mice were housed in the Animal Facility of the University of Crete (Department of Biology), in rooms with controlled light cycles (12L: 12D, lights on at 0600 hr). Non pregnant uteri were collected from sexually mature female mice (6 to 8 weeks old) during the three phases of the menstrual cycle (anoestrus, proestus, and oestrus). Pregnant uteri were collected on days 2–5 of gestation. For the pre implantation stages (days 2 and 3 of pregnancy), female mice were supervolulated by receiving i.p. 5 IU of pregnant mare serum (PMS, Sigma, St. Louis) at 1400 hr and 46 hr later 5 IU human chorionic gonadotropin (hCG, Sigma). Five hours after the last injection, the female mice were individually caged with proven male breeders and examined for the presence of vaginal plug the following morning, which was considered as day zero of pregnancy. Before including the uteri in the study, the presence of embryos in the oviduct (day 2 of pregnancy) and uterus (day 3 of pregnancy) was verified. For the collection of uteri on days 4 and 5 of pregnancy, female mice were only checked for oestrus and were individually caged overnight with proven male breeders.

### Antibodies

Rat anti-mouse IgG2a monoclonal antibodies to the growth factors GM-CSF (sensitivity 5 μg/ml), IL-3 (sensitivity 3 μg/ml), IL- 10 (sensitivity 0.14 U/ml), and IL-6 (sensitivity 50 pg/ml), purchased from Endogen Inc. (Cambridge, MA), were used at the concentration of 1 μg/ml.

### Blastocyst Collection

At 1100 hr on the third day of pregnancy, uteri were isolated in M16 medium (Sigma) and supplemented with 5% Fetal Calf Serum (FCS, Gibco, Grand Island, NY). After removing the excess fat and mesentery, uteri were flushed out using a torn Pasteur pipette. Blastocysts collected in M16 medium were fixed in a paraformaldehyde solution (3% PFA, Sigma). Blastocysts were examined for the intracellular presence of GM-CSF, IL-3, IL-10, and IL-6 by immunofluorescence.

### Cell Cultures

Uteri were put in a single cell suspension and cultured in DMEM medium supplemented with 10% FCS (Gibco, at a concentration of $1 \times 10^6$ cells/ml in 6-well plates (Nunc, Kampstrup, Denmark) for 2 to five days. Culture supernatant was collected, centrifuged, and used in ELISA experiments, and at the same time, uterine cells were submitted to immunofluorescence staining.

### Enzyme-Linked Immunoassays

Supernatant collected from days 2–4 of uterine cell cultures were used at the dilution of 1:2 in carbonate buffer pH 9.6. These were coated in 96-well flat bottom plates (Sarstedt, Newton, NC), incubated overnight at 4°C, and washed four times in 5% Tween-20. The remaining protein free sites in the plate were blocked by 2% PBS-BSA solution after an inculation of 2 hr at room temperature. After washing four times, 100 μl of test antibodies diluted in 0.1%

PBS-BSA were added and incubated for 1 hr at room temperature. Extensive washing of the plate was followed by addition of 100 µl of goat anti-mouse IgG coupled to horseradish peroxidase (1: 1,000 dilution, Sigma), which were incubated for 1 hr at room temperature, in the dark. Finally, the reaction was developed by adding 100 µl/well of tetramethyl benzidine-$H_2O_2$ (Sigma) for 20 min. The enzymatic reaction was stopped with 50 µl $H_2SO_4$ (4N). Optical density (OD) was measured at 450 nm using a Titertec ELISA photometer (Digiscan, ASYS Hitech GmbH, Engendorf, Austria). Each experiment was repeated at least four times. The results are expressed as net OD values (± SD, calculated from four or more experiments), where control OD (coating buffer or DMEM culture medium, where appropriate) was substracted from test values.

### Indirect Immunofluorescence

Immunofluorescent staining of cytoplasmic growth factor production was performed as described by Sander et al. (1991). Uterine cells were scraped off the culture plates and fixed with ice-cold paraformaldehyde (4% in PBS) for 5 min. After washing, the cells were incubated for 30 min at room temperature with specific antibodies diluted in HBSS-Saponin solution (HBSS: Gibco, 0.01 M Hepes: Gibco, 0.1% Saponin: Sigma). After washing in PBS-Saponin, the cells were incubated with rabbit anti-rat IgG antibody FITC-conjugated for 30 min at room temperature. The cells were washed, fixed in 25% glycerol, mounted on slides, and examined for cytoplasmic staining using a Leitz fluorescent microscope. Blastocysts were incubated in droplets containing the specific primary antibodies, washing medium, or secondary FITC-conjugated antibody.

### Immunoperoxidase Staining

Frozen uteri were mounted onto the cryostat using M-1 embedding matrix (U.K. Lipshaw, Germany) and 6–8 µ sections were cut using a Leitz cryostat (Model 1720), The sections were collected on preheated slides, fixed in acetone for 10 min, and kept at −20°C until used. For the immunoperoxidase staining, we followed the technique described by Willingham (1990). The sections were then counterstained with Gill's hematoxylin, dehydrated, mounted, and examined using a light microscope.

***Acknowledgements***

We thank Dr. S. Vassiliadis for critical review of the manuscript. This work was supported by University of Crete funds.

***References***

Athanassakis, I., Bleackley, R. C., Paetkau, V., Guilbert, L. J., Barr, P. J., and Wegmann, T. G. (1987). The immunostimulatory effect of T Cells and the T cell lymphokines on murine fetally-derived placental cells. *J. Immunol.* **138**: 37–44.

Chaouat, G., Menu, E., Clark, D. A., Dy, M., Minkawski, M., and Wegmann, T. G. (1990). Control of fetal survival in CBA × DBA/2 mice by lymphokine therapy. *J. Reprod. Fertil.* **89**: 447–458.

Chaouat, G., Melliani, A. A., Martal, J., Raghupathy, R., Elliot, J., Mosmann, T., and Wegmann T. G. (1995). IL-10 prevents naturally occurring fetal loss in the CBA × DBA/2 mating combination, and local defect in IL-10 production in this abortion-prone combination is corrected by *in vivo* injection of IFN-τ. J. *Immunol.* **154**: 4261–4268.

Clark D. A., Chaouat, G., Mogil, R., and Wegmann, T.G. (1994). Prevention of spontaneous abortion in DBA/2-mated CBA/J mice by GM-CSF involves CD8+ T cell dependent suppression of natural effector cell cytotoxicity against trophoblast target cells. *Cell. Immunol.* **154**: 143–152.

Garcia-Lloret, M. I., Morrish, D. W., Wegmann, T. G., Honore, L., Turner, R. A., and Guilbert, L. J. (1994). Demonstration of functional cytokine/placental interactions: CSF-1 and GM-CSF stimulate human cytotrophoblast differentiation and peptide hormone secretion. *Exp. Cell Res.* **214**: 46–54.

Haimovici, F., Hill, J. A., and Anderson, D. J. (1991). The effects of soluble products of activated lymphocytes and macrophages on blastocyst implantation events *in vitro. Biol. Reprod.* **44:** 69–75.

Heinrich, P.C., Castell, J.V., and Andus, T. (1990). Interleukin-6 and the acute phase response. *J. Biochem.* **265**: 621–636.

Hill, J. A., and Anderson, D. J. (1989). The effects of lymphokines and monokines on sperm fertilising ability in the zona-free hamster egg penetration test. *Am. J. Obstet. Gynecol.* **160**: 1154–1159.

Hill, J. A., and Anderson, D. J. (1991). Tumor necrosis factor-α and sperm function? *Fertil. Steril.* **56**: 332–339.

Makrigiannakis, A., Zoumakis, E., Margioris, A., Theodoropoulos, P., Stournaras, C., and Gravanis, A. (1995). The corticotropin-realising hormone in normal and tumoral epithelial cells of human endometrium. *J. Clin. Endocrinol. Metabol.* **80**: 185–189.

Prahala, R. H., and Wira, C. R. (1995). Sex hormone and IL-6 regulation of antigen presentation in the female reproductive tract mucosal tissues. *J. Immunol.* **155:** 5566–5573.

Sander, B., Andersson, J., and Andersson, U. (1991). Assessment of cytokines by immunofluorescence and the paraformaldehyde-saponin procedure. *Immunol. Rev.* **119:** 65–93.

Tartakovsky, B., Goldstein, O., and Brosh, N. (1991). Colony stimulating factor-1 blocks early pregnancy in mice. *Biol. Reprod.* **44:** 906–912.

Tatrakovsky, B., and Ben-Yair, E. (1991). Cytokines modulate pre-implantation development and pregnancy. *Dev. Biol.* **146:** 345–352.

Willingham, M. C. (1990). Immunocytochemical methods: Useful and informative tools for screening hybridomas and evaluating antigen expression. *Focus* **2:** 62–67.

